# Yellow Pygmy Rice Rat (*Oligoryzomys flavescens*) and Hantavirus Pulmonary Syndrome in Uruguay

**DOI:** 10.3201/eid0907.030044

**Published:** 2003-07

**Authors:** Adriana Delfraro, Mario Clara, Lorena Tomé, Federico Achaval, Silvana Levis, Gladys Calderón, Delia Enria, Mario Lozano, José Russi, Juan Arbiza

**Affiliations:** * Facultad de Ciencias de la Universidad de la República, Montevideo, Uruguay; † Instituto Nacional de Enfermedades Virales Humanas “Dr. Julio I. Maiztegui,” Pergamino, Buenos Aires, Argentina; ‡ Universidad Nacional de Quilmes, Bernal, Argentina; § Ministerio de Salud Pública, Montevideo, Uruguay

**Keywords:** hantavirus, reservoir host, *Oligoryzomys flavescens*, Uruguay, research

## Abstract

During 5,230 trapping nights, 672 small mammals were trapped in the areas where most hantavirus pulmonary syndrome (HPS) cases occur in Uruguay. Yellow pygmy rice rats (*Oligoryzomys flavescens*) were the only rodents that showed evidence of antibodies to hantavirus, with a seroprevalence of 2.6%. The rodents were trapped in all the explored environments, and most of the seropositive rodents were found in habitats frequented by humans. Nucleotide sequences were obtained from four HPS case-patients and four yellow pygmy rice rats of the M genome segment. Sequence comparison and *phylogenetic analysis showed that rodent-borne viruses and viruses from three HPS* case-patients form a well-supported clade and share a 96.4% identity with the previously characterized Central Plata hantavirus. These results suggest that yellow pygmy rice rat *(O. flavescens)* may be the host for Central Plata, a hantavirus associated with HPS in the southern area of Uruguay.[

The family *Bunyaviridae* consists of five genera. Viruses in the *Hantavirus* genus are unique among them because all members (except Thottapalayam virus) are rodent-borne. Viruses in the other four genera are arthropod-borne. Hantavirus pulmonary syndrome (HPS) was first identified in the United States in 1993. The discovery of the outbreak was followed by the identification of Sin Nombre virus (SNV) as the primary etiologic agent of HPS (*1*). Since these findings, many countries in the Americas have identified cases and outbreaks of this syndrome, and several other related viruses (New World hantaviruses) have been recognized (*2*–*9*).

New World hantaviruses are carried by different species of sigmodontine and arvicoline rodents (*Muridae*). Indeed, genetic diversity and geographic distribution of these viruses are related to the genetic diversity, geographic distribution, and phylogenetic history of their rodent hosts. In South America, studies of the correlation between rodent hosts and indigenous hantaviruses are complicated by the great diversity of sigmodontine rodents in this area. Also, the sympatric distributions between the different species of sigmodontine rodents in South America provide opportunities for spillover infections and host-switching events (*10*).

In Uruguay, the first evidence of the circulation of these viruses came from a study of serum specimens collected from blood donors between 1985 and 1987 that showed a seroprevalence of 1%, as measured by indirect fluorescent antibody (IFA) test using Hantaan and Seoul antigens (*11*). Since 1997, the Ministerio de Salud Pública, through the Departamento de Laboratorios, began the surveillance and diagnosis of HPS. In 2000 Padula et al. (*12*) reported partial sequences (G1 and G2 glycoprotein) derived from two HPS cases that occurred in Uruguay in 1997 and 1999. These viruses clustered within a previously reported lineage named Central Plata.

Knowledge about small mammal communities and habitat preferences is limited in Uruguay. However, some studies about systematic distribution, reproduction, and cytogenetic aspects have been published (*13*–*17*). Research regarding the distribution and habitat preferences of the *Muridae* family in Uruguay is currently being conducted (*18*–*20*).

The purpose of this study was to identify the carrier rodents of hantavirus in Uruguay and their potential association with HPS cases, to determine the prevalence of infection in different habitats, and to begin to genetically characterize the hantaviruses recovered from these rodents.

## Material and Methods

### HPS Case Identification

National surveillance for HPS was reinforced when a case definition was established by the Ministerio de Salud Pública in 1997. An HPS case was suspected in a previous healthy person with an acute febrile illness (temperature >38°C), associated with dyspnea, acute respiratory distress syndrome with pulmonary noncardiogenic edema, or interstitial bilateral infiltrates, hypotension or shock, elevated leukocyte count, and thrombocytopenia (*21*). A case of HPS was confirmed when, in addition to clinical illness, circulating specific hantavirus immunoglobulin (Ig) M was detected.

Human serum samples were tested for the presence of IgM and IgG antibodies with an enzyme-linked immunosorbent assay (ELISA) developed by MRL (Hantavirus ELISA IgM and Hantavirus ELISA IgG, MRL Diagnostics, Cypress, CA). The test was used to screen patients, and in every case, the results were confirmed by retesting the specimens by an in-house enzyme immunoassay with a recombinant nucleocapsid antigen specific to Andes virus, according to the procedure developed by Padula et al. (*12*).

### Site Selection

Rodent sampling was conducted at the most likely sites of infection for known HPS case-patients and included the places where the person had lived or worked in the 6 weeks before onset of symptoms and nearby natural habitats. The trapping sites were classified as 1) domestic and peridomestic, including all sites in the immediate vicinity of houses, sheds, gardens, road borders, and fence lines, and 2) rural natural ecosystems and agro-ecosystems, including representative habitats of each area such as open fields, cultivated areas, wetlands, shrublands, brook borders, natural forests, and artificial woods (planted by humans) (Table 1).

**Table 1:** Trapping efficiency/environment/species in the total of captures^a^

Env	TN	C	E%	Md	Of	Od	St	Aa	No	Hb	Cl	Mm	Rr	Ca
NW	288	27	9.4	-	1	7	10	6	2	-	1	-	-	-
RB	1,010	172	17.0	-	55	4	24	11	14	-	1	63	-	-
PD	1,420	44	3.1	1	19	1	7	-	2	-	-	11	-	3
WE	265	14	5.2	-	2	-	8	-	2	2	-	-	-	-
BB	680	57	8.4	1	11	1	23	4	10	-	-	7	-	-
AG	190	50	26.3	-	9	6	9	2	6	-	1	17	-	-
SH	1,198	268	22.4	1	93	2	117	11	13	-	-	30	-	1
AW	179	40	22.3	1	4	1	-	-	3	-	-	29	2	-
T	5,230	672	12.8	4	194	22	198	34	52	2	3	157	2	4
Sp%				0.6	28.9	3.3	29.5	5.1	7.7	0.3	0.4	23.4	0.3	0.6

^a^Env, environments; TN, trapping nights; C, captures; E, efficiency; NW, natural woods; RB, road borders; PD, peridomestic; WE, wetlands; BB, brook borders; AG, agroecosystems; SH, shrublands; AW, artificial woods; T, totals; Sp%, species %; *Md*, *Monodelphis dimidiate*; *Of*, *Oligoryzomys flavescens*; *Od, O. delticola; St*, *Scapteromys tumidus;* *Aa*, *Akodon azarae; n*, *Necromys obscurus; Hb, Holochilus brasiliensis*; *Cl*, *Calomys laucha*; *Mm, Mus musculus*; *Rr, Rattus rattus*; *Ca*, *Cavia aperea*.

**Figure 1:**
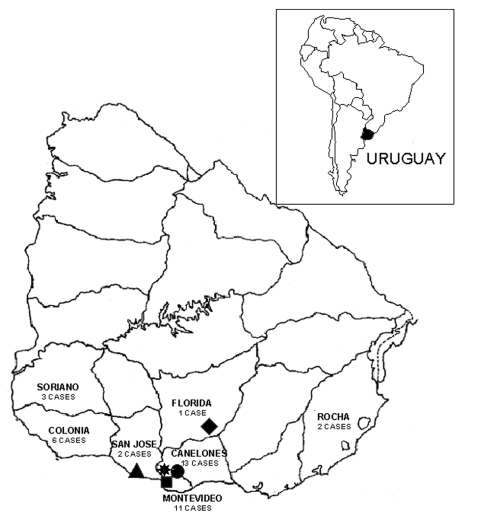
Locations of capture sites and enzyme-linked immunosorbent assay–confirmed hantavirus pulmonary syndrome case-patients in Uruguay. Capture sites: red triangle = Puntas de Valdéz, green diamond = Piedritas, blue circle = Sauce, yellow star = Cerrillos, green square = Melilla.

The trapping expeditions were performed in the following areas: Puntas de Valdéz (34°32′S/56°36′W) and Piedritas (34º20′S/55°39′W) (one expedition each); Cerrillos (34°38′S/56°19′W), Melilla (34º44′S/56°16′W), and Sauce (34°35′S/56°08′W) (two expeditions each) (Figure 1). The geographic area covered by the trapping expeditions corresponded to areas where 16 HPS cases occurred in Montevideo and Canelones, two cases occurred in San José, and one case occurred in Florida. The other 19 cases were dispersed in the southern half of Uruguay, and for some of them, the probable site of infection was not clearly identified.

The landscape of Canelones, rural Montevideo, Florida, and San José shows cultivated areas, stubble areas, shrublands in the territories abandoned by rural people, range lands, natural and artificial woodlands, small wetlands, and small brooks. In recent years, many rural inhabitants have migrated to the cities; abandoned farmlands have thus been transformed into shrublands.

### Small-Mammal Trapping and Processing

Small mammals were trapped in six expeditions in the above-mentioned areas from May 1997 until September 2001. Each trapping site was sampled with Sherman live-capture traps (model LFATDG 23 cm x 8 cm x 8.5 cm) (Sherman Traps Inc., Tallahassee, FL). The number of traps depended on the available area for trap placement at each trapping site. The traps were placed at 5-m intervals in line transects, along the different environments at the trapping site. The traps were set out in the late afternoon and checked in the early morning for the next two mornings. The animals were trapped and sampled according to established biosafety guidelines (*22*). Each animal was anesthetized, and blood was collected from the retroorbital sinus. The animals were humanely killed, and their size, mass, sex, and reproductive status were recorded. Samples of liver, kidney, lung, and brain were extracted and stored in liquid nitrogen for further processing. The individual animals were tentatively identified in the field by external characteristics, and the carcasses were kept in 10% formalin. Identification was confirmed by cranial measurements and dental examination at the laboratory. All specimens were archived in the Sección Zoología Vertebrados, Facultad de Ciencias, Universidad de la República, Montevideo, Uruguay.

### ELISA

Serologic testing of rodents was performed by IgG ELISA. Briefly, the IgG ELISAs were performed by coating polyvinyl chloride microtiter plates (Dynex Technologies, Chantilly, VA) overnight at 4°C with a Lechiguanas virus (LECV) antigen (inactivated, 3 M rad gamma-ray irradiation detergent-extracted lysate of Vero-E6 infected cells, with a 100% infection index controlled by indirect immunofluorescence). An uninfected Vero E6 cell culture antigen was used to determine the specificity of mouse antibodies. Unbound antigen was removed by washing three times with phosphate-buffered saline (PBS)-Tween 20, 0.1% (Sigma-Aldrich, St. Louis, MO). After blocking with PBS-Tween 20, 0.1%-dry milk 5% (37°C, 1 h), sera diluted fourfold, beginning with 1:100, were added to react with the antigen-coated wells. Bound antigen was measured by the use of a hyperimmune mouse ascitic fluid and by using goat anti–*Peromyscus leucopus* IgG (H+L) and goat-anti rat IgG (heavy- and light-chain–specific; Kirkegaard & Perry Laboratories, Gaithersburg, MD) conjugated to horseradish peroxidase. Optical densities (ODs) at 405 nm were recorded on a microplate spectrophotometer (Labsystems Multiskan EX; Thermo Labsystems, Finland, Vartaa, Finland), and the ODs of the uninfected antigen-coated well were subtracted from that of its corresponding viral antigen to yield the adjusted OD. A serum dilution was considered positive if OD was >0.2 U after adjustment. A serum titer >400 was considered positive.

### Total RNA Extraction and RT-PCR

Total RNA was extracted from lung tissue of seropositive rodents and from blood clots from HPS case-patients. Approximately 100 mg of tissue was treated with 1 mL of TRIzol reagent (GIBCO BRL, Life Technologies, Rockville, MD), according to manufacturer’s instructions. An M genome segment of the G2 glycoprotein encoding region was amplified by using reverse transcription-polymerase chain reaction (RT-PCR) and specific oligonucleotides as previously described by Levis et al. (*4*).

RT was carried out using MMLV reverse transcriptase (GIBCO BRL) and the oligonucleotide 3348(-) (5′CTGTCCAGATTTAGTGTTCCA 3′). cDNA was then precipitated with NaAc (ICN Biomedicals, Costa Mesa, CA) 3 M pH 5.6, ethanol (Merck Química Argentina, Buenos Aires, Argentina), and lineal polyacrylamide 2.5 μg/μL (ICN Biomedicals), and resuspended in 20 μL of double distilled water. Two microliters of first-strand cDNA was used in the PCR reaction. Two rounds of PCR were performed by using Taq DNA polymerase (GIBCO BRL). The first round was performed with oligonucleotides 3348 (-) and 2765 (+) (5′CTGTATGTGAGTACCAAG 3′), and the second round (heminested) was performed with 1 μL of first-round reaction and oligonucleotides 3221 (-) (5′TCAGAAGAGCAGTCAGTGTCATG 3′) and 2765 (+), giving a 456-nucleotide (nt) fragment. PCR fragments were visualized on ethidium bromide 1.5% agarose gels.

### Sequencing and Phylogenetic Analysis

The PCR fragments obtained from rodent and HPS case-patient samples were purified for further sequencing by using the Concert rapid gel extraction system (GIBCO BRL) or QIAquick gel extraction kit (QIAGEN Inc., Valencia, CA). Nucleotide sequencing was conducted by using the oligonucleotide 2765(+) and an ABI 377 Genetic Analyser (PE Applied Biosystems, Inc., Foster City, CA).

Alignment of sequences was done by using CLUSTALX (1.5) (*23*). Phylogenetic analyses and sequence comparison were carried out with PAUP* 4.0b10 (*24*) and MEGA version 2.1 (*25*). Maximum parsimony analysis was carried out by using the heuristic search option. Maximum parsimony trees were searched by applying the tree bisection reconnection branch-swapping algorithm. A consensus tree was obtained through 50% majority rule consensus. For the distance-based approach, MODELTEST 3.06 (*26*) was used to establish the most suitable model of DNA substitution that best fitted our dataset, and a phylogenetic tree was obtained by using the neighbor-joining algorithm. Bootstrap analysis (*27*) was performed to estimate topologic accuracy of the trees (500 replicates), and only values >70% were considered significant.

For comparison, existing sequence data from GenBank were used: hantavirus sequences from Argentina (GenBank accession nos. AF028023 to AF028027, AF028029 to AF028063), Central Plata genotype from Uruguay (GenBank accession nos. AY101184 and AY101185), and Sin Nombre virus (L37903, isolate NMR11); the last one was used as outgroup.

## Results

### HPS Cases

From April 1997 to August 2002, 38 cases of HPS were confirmed by ELISA, with a fatality rate of 21.0%. Twenty-four (63.2%) of these cases occurred in rural or suburban areas of Montevideo and Canelones, 6 (15.7%) in Colonia, 3 in Soriano (7.9%), 2 in San José (5.3%), 2 in Rocha (5.35%), and 1 in Florida (2.6%) (Figure 1). As of August 2002, HPS cases in Uruguay had occurred in the southern half of the country.

### Distribution of Rodents by Species and Capture Site

During 5,230 trap-nights, 672 small mammals were collected (trap success = 12.8%). The trapped small mammals belonged to two families (*Muridae* and *Caviidae*) within the order Rodentia and one family (*Didelphidae*) in the order Didelphimorphia. The mammals belonged to 11 species, 75.1% of the captured animals corresponded to the Sigmodontinae subfamily, 23.7% to the Murinae subfamily, 0.6% to the *Caviidae* family, and 0.6% to the *Didelphidae* family (Table 1).

Captures and percentage of trap success by habitats were as follows: natural woodlands, 27 (9.4%) of 288; road borders, 172 (17.0%) of 1,010, peridomestic areas, 44 (3.1%) of 1,420; wetlands,14 (5.2%) of 65; brook borders, 57 (8.4%) of 680; agroecosystems, 50 (26.3% of 190; shrublands, 268 (22.4%) of 1,198; and in artificial woodlands, 40 (22.3%) of 179 (Table 1).

The most common captured small mammals were the following: swamp rats (*Scapteromys tumidus)*, 198 (29.5%); yellow pygmy rice rats (*Oligoryzomys flavescens),* 194 (28.9%); and house mice (*Mus musculus),* 157 (23.4%) (Table 1). No sigmodontine rodents were found inside the houses, where only house mice and black rats (*Rattus rattus)* were found. Yellow pygmy rice rats were found in areas of human disturbance such as peridomestic areas, agroecosystems, road borders, and shrublands. We found that the trapping success in these sites was higher than in natural areas. As shown in Table 1, yellow pygmy rice rats were found in all of the habitats where traps were set.

### Screening for Hantavirus Infection of Rodents

Serum specimens collected from rodents were screened for IgG antibodies to LECV by ELISA. As mentioned above, 672 small mammals were trapped in six areas where HPS cases occurred between 1997 and 2001. Anti-LECV antibodies were detected in five rodents (*O. flavescens* ) from four different locations. Absorbances with LECV antigen of positive samples screened at 1:400 dilution were at least fourfold the absorbance of the negative control. Further titration showed that three samples had titers >1:1,600, and one had a titer >1:6,400 (Table 2). The proportion of positive rodents in the different localities ranged from 2.1% to 2.9%. Piedritas was the only locality where no antibody-positive rodents were recorded (Table 3).

**Table 2:** Hantavirus-seropositive rodents found in the different geographic areas where captures were performed

Sample	Rodent species	Geographic area	Habitat	Antibody titer (arbitrary units)
U89^a^	*Oligoryzomys flavescens*	Puntas de Valdéz (San José)	Road border	1,600	
SA63^a^	*O. flavescens*	Sauce (Canelones)	Peridomestic	1,600
Ce20^a^	*O. flavescens*	Melilla (Montevideo)	Peridomestic	>6,400
Ce22^a^	*O. flavescens*	Melilla (Montevideo)	Shrublands	1,600
Ce155^a^	*O. flavescens*	Cerrillos (Canelones)	Shrublands	400

^a^Specimens deposited at the Specimen Collection of the Sección Zoología Vertebrados, Facultad de Ciencias, Universidad de la República, Montevideo, Uruguay with the following numbers: U89=ZVC-M2154, SA63=ZVC-M2155, Ce20=ZVC-M2156, Ce22=ZVC-M2157, and Ce155=ZVC-M2158.

**Table 3:** Small mammals trapped in the different sites, number and % of positives^a^

Area	*Md*	*+/%*	*Of*	*+*/%	*Od*	*+/%*	*St*	*+/%*	*Aa*	*+/%*	*No*	*+*/%	*Hb*	*+/%*	*Cl*	*+/%*	*Mm*		*+/%*	*Rr*	*+/%*	*Ca*	*+/%*	Total
PV	-	-/-	38	1/2.6	-	-/-	-	-/-	10	0/0	-	-/-	-	-/-	2	0/0	81	0/0		-	-/-	-	-/-	131
Ce	-	-/-	47	1/2.1	9	0/0	52	0/0	4	0/0	17	0/0	2	0/0	-	-/-	23	0/0		-	-/-	-	-/-	153
Me	-	-/-	67	2/2.9	8	0/0	37	0/0	-	-/-	17	0/0	-	-/-	1	0/0	10	0/0		-	-/-	3	0/0	297
Pi	-	-/-	4	0/0	-	-/-	8	0/0	14	0/0	-	-/-	-	-/-	-	-/-	2	0/0		-	-/-	-	-/-	28
Sa	3	0/0	42	1/2.4	1	0/0	92	0/0	5	0/0	18	0/0	-	-/-	-	-/-	12	0/0		-	-/-	-	-/-	173
Ca	1	0/0	-	-/-	-	-/-	9	0/0	1	0/0	-	-/-	-	-/-	-	-/-	29	0/0		2	0/0	1	0/0	43
Total	4	0/0	194	5/2.6	22	0/0	198	0/0	34	0/0	52	0/0	2	0/0	3	0/0	157	0/0		2	0/0	4	0/0	672

^a^*Md,* *Monodelphis dimidiate; Of, Oligoryzomys flavescens; Od, O. delticola; St, Scapteromys tumidus*, *Aa,* *Akodon azarae*, *No*, *Necromys obscurus; Hb*, *Holochilus brasiliensis*; *Cl,* *Calomys laucha; Mm, Mus musculus;* *Rr* ,*Rattus rattus; Ca, Cavia aperea;* PV, Puntas de Valdéz; Ce, Cerrillos; Me, Melilla; Pi, Piedritas, Sa, Sauce; Ca, Canelones. *O. flavescens, S. tumidus,* and *M. musculus* were the most frequently captured rodents.

### Total RNA Isolation, RT-PCR, and Sequence Analysis

Total viral RNA was extracted from the lungs of the five seropositive yellow pygmy rice rats and blood clots from four case-patients. RT of viral RNA and PCR amplification of a 456-nt fragment of the G2 glycoprotein–encoding region of the virus M genome segment (bases corresponding to LECV 2,805–3,215) and nucleotide sequences were obtained from four rodent samples and four human blood clots. Amplified DNA was not recovered from the rodent sample CE155, which had the lower antibody titer (Table 3)*.* A 292-nt segment (LECV 2,815–3,106 G2 glycoprotein–encoding region) was used for further comparison and phylogenetic analysis.

### Sequence Comparison and Phylogenetic Analysis

**Figure 2:**
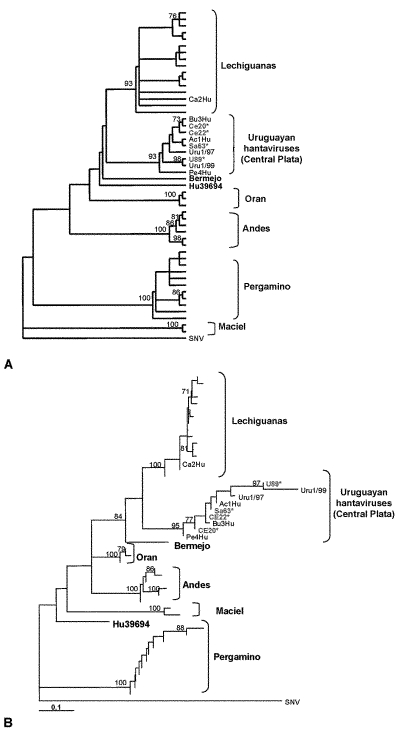
A: Maximum parsimony phylogenetic tree B: Distance-based phylogenetic tree. The tree was built under the Tamura-Nei model of DNA substitution with estimation of the shape parameter of the gamma distribution (*28*). This model and the associated parameters resulted from testing our dataset with the program MODELTEST 3.06 (*26*). Both trees include Argentinean and Uruguayan hantaviruses from hantavirus pulmonary syndrome (HPS) case-patients and rodents. Hantavirus sequences from HPS case-patients in Uruguay: Ac1Hu, Ca2Hu, Bu3Hu, Pe4Hu, Uru1/97, Uru1/99. Hantavirus sequences from yellow pygmy rice rats: U89, Sa63, Ce20, Ce22. Sin Nombre virus was used as outgroup.*Specimens deposited at the Specimen Collection of the Sección Zoología Vertebrados, Facultad de Ciencias, Universidad de la República, Montevideo, Uruguay, with the following numbers: U89=ZVC-M2154, SA63=ZVC-M2155, Ce20=ZVC-M2156, Ce22=ZVC-M2157, and Ce155=ZVC-M2158.

For phylogenetic comparisons, a 292-nt fragment of the M gene from lung RNA of four yellow pygmy rice rats (GenBank accession nos. AY204677 to AY204680), as well as clot RNA from four HPS case-patients (GenBank accession nos. AF283896 to AF283899) was used. These nucleotide sequences were compared with the equivalent region of published hantavirus sequences. Phylogenetic analysis indicated that two previously known hantavirus genotypes are circulating in Uruguay: Central Plata and LEC (Figure 2).

By both maximum parsimony and distance-based analysis, the four sequences recovered from Uruguayan yellow pygmy rice rats were closely related to each other and formed a monophyletic group with the hantavirus sequences derived from three HPS case-patients from Canelones and Montevideo and two HPS case-patients from the same geographic area, previously characterized as Central Plata. This clade was supported by high bootstrap values (Figure 2A,B). Comparison of these sequences at the nucleotide level showed 96.4% identity. The most closely related genotype was LEC, with 87.9% identity, followed by Bermejo (85.0%), Orán (83.1%), Andes (82.5%), and Hu39694 (82.1%). The less-related genotypes of the Argentinean hantaviruses were Maciel (79.3%) and Pergamino (78.6%). One viral sequence from an HPS case-patient in Soriano clustered into LEC genotype.

## Conclusion

Most HPS cases were in rural and suburban areas of Montevideo and Canelones (24 of 38 cases) (Figure 1) in southern Uruguay. Rodent sampling was conducted at the most likely sites of infection for known HPS case-patients.

The trapping success rate was higher in the environments influenced by humans (agroecosystems, road borders, shrublands, artificial woods, peridomestic areas) than in the natural areas (natural woods, wetlands, brook borders): 574 (85.4%) of trapped individual animals were captured in environments influenced by humans, and 98 (14.6%) were captured in natural environments. Swamp rats, yellow pygmy rice rats, and house mice were the most frequently trapped species. Of 44 (3.1%) rodents trapped in peridomestic environments, 19 (43.2%) were yellow pygmy rice rats (Table 1). Five seropositive yellow pygmy rice rats were captured in modified environments: one was captured along a road border, two were captured in peridomestic environments, and two were captured in shrublands, at <150 m from homes. Three of five seropositive rodents were therefore trapped in environments frequented by humans (road borders and peridomestic environments). These findings could indicate an increased risk for infection for human inhabitants.

Yellow pygmy rice rats were the only rodents that showed evidence of antibodies to hantavirus, with a prevalence of 2.6%. Researchers have found that hantavirus seroprevalence in rodents may vary widely, according to the season, geographic area, altitude, and rodent species analyzed (*29*–*33*). We found that the percentage of seropositive rodents (2.6%) is the same as encountered in the central zone of Argentina (2.6%) (*32*), although the habitats are not similar to the southern area of Uruguay. In Uruguay, we found that only yellow pygmy rice rats were antibody positive, while in central Argentina seropositive yellow pygmy rice rats, Azara’s field mice (*Akodon azarae)*, dark mice (*Necromys benefactus*), and small water rats (*Holochilus brasiliensis*) were found (*2*–*4*). In the different locations in Uruguay, seroprevalence was similar, ranging from 2.1% to 2.9%. In Piedritas, where no positive rodents were found, only four yellow pygmy rice rats were trapped. All seropositive rodents in Uruguay were adult males, which is consistent with horizontal transmission and in accordance with the findings of several authors (*30*–*32*).

The phylogenetic analysis on a 292-nt region of the M segment showed that these rodent sequences clustered together with those from five Uruguayan HPS case-patients from the same geographic area (Canelones and Montevideo); these data suggest that the yellow pygmy rice rat can be considered as the putative reservoir host for Central Plata hantavirus in this region of Uruguay. This study also showed the circulation of LEC genotype in the western location of Soriano, 250 km from Montevideo, separated from the Argentinean central HPS-endemic area by the Uruguay River. This virus shared a 99% identity at the nucleotide level with LEC genotype.

Phylogenetic analysis shows that the genotype Central Plata recovered from rodents and HPS case-patient from Canelones, San José, and Melilla is phylogenetically distinct from (although related to) the previously described LEC genotype, whose reservoir host in Argentina is also the yellow pygmy rice rat. Hantaviruses have been associated with subspecies of closely related rodents: Sin Nombre–like hantaviruses with mice from the genus *Peromyscus* (*34*) and Andes virus recovered in southwestern Argentina and Orán virus in northwestern Argentina, both recovered from long tailed pygmy rice rats (*O. longicaudatus*) (4). Recent studies have shown that these two rodent populations differ with respect to their mitochondrial DNA (*10*). This fact raises the question of whether rodents morphologically identified as *O. flavescens* in Uruguay are indeed a different subspecies of *O. flavescens* in Argentina. Further experiments will be needed to identify both the interspecific and intraspecific phylogenetic relationships of *O. flavescens* in these regions.
